# Vendors’ perspectives on AI implementation in medical imaging and oncology: a cross-sectional survey

**DOI:** 10.1007/s00330-025-12013-1

**Published:** 2025-09-23

**Authors:** Nikolaos Stogiannos, Emily Skelton, Kicky Gerhilde van Leeuwen, Sally Edgington, Susan Cheng Shelmerdine, Christina Malamateniou

**Affiliations:** 1 CRRAG research group, Division of Radiography, Department of Allied Health, School of Health and Medical Sciences, City St George’s University of London, University of London, London, UK; 2 Magnitiki Tomografia Kerkiras, Corfu, Greece; 3 European Federation of Radiographer Societies, Cumiera, Portugal; 4 Romion Health, Utrecht, The Netherlands; 5 Health AI Register, Utrecht, The Netherlands; 6 AXREM, London, UK; 7 Department of Clinical Radiology, Great Ormond Street Hospital for Children NHS Foundation Trust, London, UK; 8 University College London Great Ormond Street Institute of Child Health, London, UK; 9 NIHR Great Ormond Street Hospital Biomedical Research Centre, Bloomsbury, London, UK; 10 European Society of Medical Imaging Informatics, Vienna, Austria; 11 Department of Neuroimaging, King’s College London, London, UK

**Keywords:** Artificial intelligence, Industry, Diagnostic imaging, Radiation oncology

## Abstract

**Objectives:**

To explore the perspectives of AI vendors on the integration of AI in medical imaging and oncology clinical practice.

**Materials and methods:**

An online survey was created on Qualtrics, comprising 23 closed and 5 open-ended questions. This was administered through social media, personalised emails, and the channels of the European Society of Medical Imaging Informatics and Health AI Register, to all those working at a company developing or selling accredited AI solutions for medical imaging and oncology. Quantitative data were analysed using SPSS software, version 28.0. Qualitative data were summarised using content analysis on NVivo, version 14.

**Results:**

In total, 83 valid responses were received, with participants having a global distribution and diverse roles and professional backgrounds (business/management/clinical practitioners/engineers/IT, etc). The respondents mentioned the top enablers (practitioner acceptance, business case of AI applications, explainability) and challenges (new regulations, practitioner acceptance, business case) of AI implementation. Co-production with end-users was confirmed as a key practice by most (52.9%). The respondents recognised infrastructure issues within clinical settings (64.1%), lack of clinician engagement (54.7%), and lack of financial resources (42.2%) as key challenges in meeting customer expectations. They called for appropriate reimbursement, robust IT support, clinician acceptance, rigorous regulation, and adequate user training to ensure the successful integration of AI into clinical practice.

**Conclusion:**

This study highlights that people, infrastructure, and funding are fundamentals of AI implementation. AI vendors wish to work closely with regulators, patients, clinical practitioners, and other key stakeholders, to ensure a smooth transition of AI into daily practice.

**Key Points:**

***Question*** *AI vendors’ perspectives on unmet needs, challenges, and opportunities for AI adoption in medical imaging are largely underrepresented in recent research*.

***Findings*** *Provision of consistent funding, optimised infrastructure, and user acceptance were highlighted by vendors as key enablers of AI implementation*.

***Clinical relevance*** *Vendors’ input and collaboration with clinical practitioners are necessary to clinically implement AI. This study highlights real-world challenges that AI vendors face and opportunities they value during AI implementation. Keeping the dialogue channels open is key to these collaborations*.

**Graphical Abstract:**

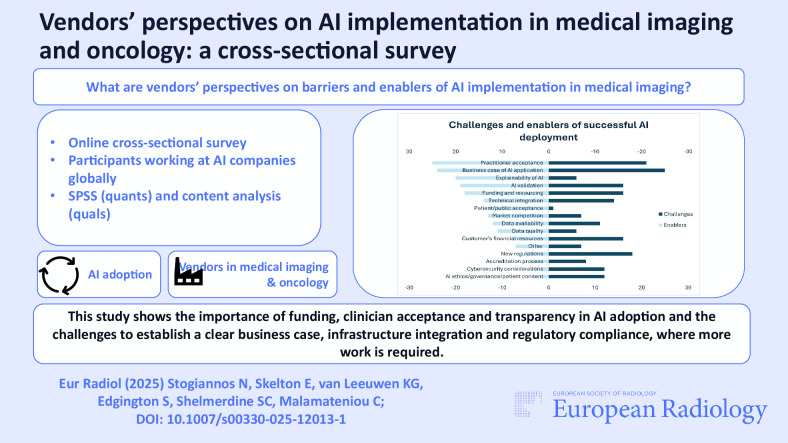

## Introduction

Artificial Intelligence (AI) in medical imaging and oncology stands at a critical juncture. Recent regulatory developments in the European Union (EU) [1], United Kingdom (UK) [2] and globally [3] are reshaping governance and, consequently, practice. While these changes aim to ensure responsible innovation and patient safety, concerns have been raised about potential barriers to implementation due to the complexity of legislation and the inhomogeneity of the AI landscape.

First, it is important to define AI implementation; this term refers to the integration of AI-enabled technologies into the processes and decision-making of an organisation [4], and it is suggested that AI implementation is a multifactorial and complex process, consisting of both societal and technical aspects [5]. AI implementation is often used interchangeably with other terms, that have similar but not the same definition, such as deployment, integration, adoption, and other terms.

Some argue that increased regulation may stifle innovation by imposing additional requirements on health tech vendors [6]; conversely, others state that well-designed regulations can accelerate the safe and effective implementation of AI by providing a clear roadmap for safe clinical practice [7]. While these frameworks and governance structures are designed to ensure patient safety and support the effective use of AI by clinicians, there is limited research on how they impact the route to market and deployment practices and how AI vendors perceive and experience these regulations. These AI vendors, ranging from large manufacturers to small and medium-sized enterprises, are key drivers for innovation in the healthcare industry by contributing substantial “know-how” and financial investment into new products and thus, understanding their viewpoint is crucial for fostering collaborations and facilitating the translation of the most promising tools from ‘code to clinic’ to ultimately benefit patient care.

The aim of this study is to understand the perspectives of vendors working in the field of AI implementation in medical imaging and oncology. By providing a platform for vendors to voice their views about what enablers and perceived challenges they face, as well as their suggestions for improvement, we hope to contribute to the ongoing dialogue surrounding responsible integration and adoption of AI, as well as to enable a collaborative effort in this evolving landscape, for positive change and patient benefit.

## Materials and methods

### Ethics

Ethics approval was provided by the City, St George’s University of London Research Ethics Committee (ref: ETH2324-2242). Participation in the survey was voluntary and without offering incentives. Respondents were asked to provide informed consent, via the online survey, for use of their responses in research outputs stemming from this work. Collected responses were anonymous (including IP addresses) and could therefore not be withdrawn.

### Study design

This is an observational, cross-sectional study. The Checklist for Reporting Results of Internet E-Surveys (CHERRIES) [8] and the Strengthening the Reporting of Observational Studies in Epidemiology (STROBE) guidelines [9] were used.

### Survey design

The survey was first developed through initial discussions with industry representatives, to ensure it was grounded on current perspectives, needs and concerns of the intended population. It was then refined after consecutive meetings and discussions among the multidisciplinary research team, the members of which had prior experience in working with different AI vendors in medical imaging or oncology research projects in different countries in Europe, ensuring diversity of expertise. The survey was finally enriched based on evidence-based practice [10–14], and all survey items were created in the light of best practices and themes, to ensure alignment with recent evidence. Prior to widespread dissemination, the survey was initially piloted by five medical imaging and oncology researchers with diverse backgrounds (radiologists, radiographers, biomedical engineers, radiology managers) and years of experience (3–25 years) to ensure content and face validity, and to allow for necessary adjustments in content and formatting. Cronbach’s alpha was calculated as a surrogate measure for survey consistency [15].

The final survey for dissemination comprised 28 questions, including 23 closed-type questions and 5 open-ended ones, to allow more depth of perspective to be shared. The full survey is provided as Supplementary Material 1.

### Eligibility criteria

Participants were invited to complete the survey if they fulfilled the following criteria:

- over 18-years-old.
- working at a company developing or selling Food & Drug Administration (FDA)/Conformité Européenne (CE)/UK conformity assessed accredited AI tools for medical imaging and oncology.

Participants with different job roles, seniority, titles, or geographical location of their offices, deployment or operating locations were invited to participate (this was an international study).

### Survey distribution

The online survey was created using the Qualtrics platform (Qualtrics). The survey was disseminated via social media (LinkedIn), using the collective reach of the researchers’ networks (more than 40,000 followers combined reach) and via personalised emails to professional contacts within the AI industry vendors. The European Society of Medical Imaging Informatics (EuSoMII) [16] and Health AI Register [17] also promoted the survey through their social media channels. Health AI register additionally shared the survey link via direct emails to representatives of the listed companies featured on their website.

The survey remained open for two months (11th July 2024 to 10th September 2024) to maximise participation. Reminder emails to potential respondents were sent every 3 weeks to encourage engagement.

### Data analysis

Before analysis, the survey responses were cleared of all invalid entries. A valid entry was defined as one having at least one response beyond the demographic questions.

Quantitative data were analysed using descriptive statistics on the Statistical Package for Social Sciences (SPSS) software, version 28.0 (IBM Corp). Qualitative data were evaluated using content analysis [18], whereby free-text responses were coded on NVivo, version 14 (QSR International Pty LTD) and organised into categories and themes, accompanied by the related frequency trends.

## Results

Cronbach’s alpha coefficient was 0.797 for this study, which can be characterised as acceptable reliability within the Cronbach’s scale [19].

### Respondent characteristics

Of the original 137 responses received, only 83 responses were valid based on the criteria described above. It should be noted that due to survey attrition, not all questions received the same number of responses. Hence, any frequencies presented below correspond to the actual number of responses received for each separate question. The main demographic data of the respondents is shown in Table 1.

**Table 1:** Main demographic data of the respondents

Age	18–29-years-old	*n* = 11 (13.3%)
	30–39-years-old	*n* = 29 (34.9%)
	40–49-years-old	*n* = 29 (34.9%)
	50–59-years-old	*n *= 12 (14.5%)
	60–69-years-old	*n* = 2 (2.4%)
Gender	Male	*n* = 52 (62.7%)
	Female	*n* = 27 (32.5%)
	Prefer not to say	*n* = 4 (4.8%)
Professional background	Business/management	*n* = 28 (33.7%)
	Clinical practitioner	*n* = 21 (25.3%)
	Engineering	*n* = 16 (19.3%)
	Informatics	*n* = 8 (9.6%)
	Other	*n* = 10 (12%)
Years of experience	0–10 years	*n* = 56 (67.5%)
	11–20 years	*n* = 19 (22.9%)
	21–30 years	*n* = 6 (7.2%)
	> 30 years	*n* = 1 (1.2%)
Company size	Micro enterprise (1–9 employees)	*n* = 4 (5%)
	Small enterprise (10–49 employees)	*n* = 20 (25%)
	Medium enterprise (50–249 employees)	*n* = 29 (36.2%)
	Large enterprise (> 250 employees)	*n* = 27 (33.8%)

Nearly half of the respondents (*n* = 38/83; 45.8%) reported being at an advanced career level, over a third of them (*n* = 34/83; 41%) were mid-career professionals, and 12% (*n* = 10/83) of them were at the beginning of their career. A generally balanced distribution was noted across the sample regarding their prior AI education/training, since 52.5% (*n* = 42/80) of them reported having acquired prior AI training, while 47.5% (*n* = 38/80) of them had not. From those who had previous AI education/training, the majority (*n* = 21/38; 55.3%) reported previous formal education, such as postgraduate courses on Machine Learning, followed by those who acquired training provided by a company (*n* = 8/38; 21.1%), those who gained on-the-job training (*n* = 4/38; 10.5%), and those who relied on self-taught reading (*n* = 4/38; 10.5%), or conferences (*n* = 1/38; 2.6%).

**Fig. 1:**
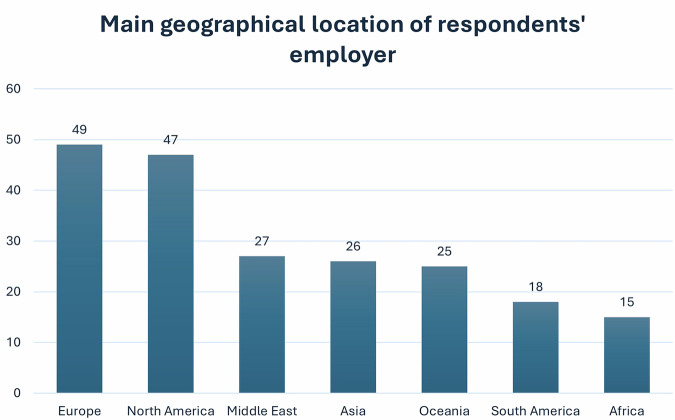
Geographical location of this study’s respondents’ employers

Most of the respondents (*n* = 65/76; 85.6%) stated that the companies they worked at provided AI training to their employees, compared to those who did not (*n* = 9/76; 11.8%). Respondents from micro enterprises reported fewer opportunities for AI training for employees (*n* = 2), compared to respondents of larger competitors (*n* = 23 for large enterprises, *n* = 26 for medium-sized companies). Figure 1 demonstrates that the companies where this study’s respondents were employed were mainly operating in Europe and North America, but the sample included participants from everywhere in the world.

### Responsible AI development and deployment

**Fig. 2:**
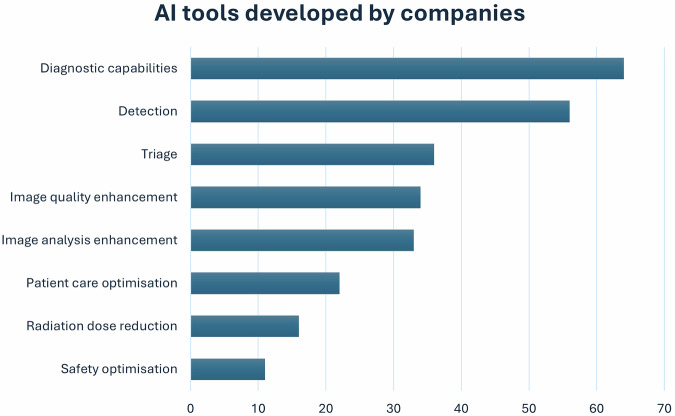
Type of AI tools developed by the companies the respondents worked at

Figure 2 summarises the different AI tools companies developed, according to the respondents (*n* = 79). Of note, some companies’ products extend beyond the above topic areas, and some companies produce more than one of these products. Most respondents (*n* = 42/71; 59.2%) confirmed that their companies employed co-production/co-creation of AI tools with end-users, compared to those who stated that they did not (*n* = 9/71; 12.7%), and those who were unsure about this (*n* = 18/71; 25.3%). A further 2.8% (*n* = 2/71) clarified that they used to involve end-users, but not always as equal partners. Thirty respondents said that their companies involved healthcare professionals or clinical collaborators in co-production. In addition, some of them employed co-production as part of their user testing and evaluation procedures. Descriptive statistics showed that respondents from larger companies reported co-production approaches being used more often than those from micro enterprises (*n* = 13 for large enterprises, compared to *n* = 2 for micro enterprises).

**Fig. 3:**
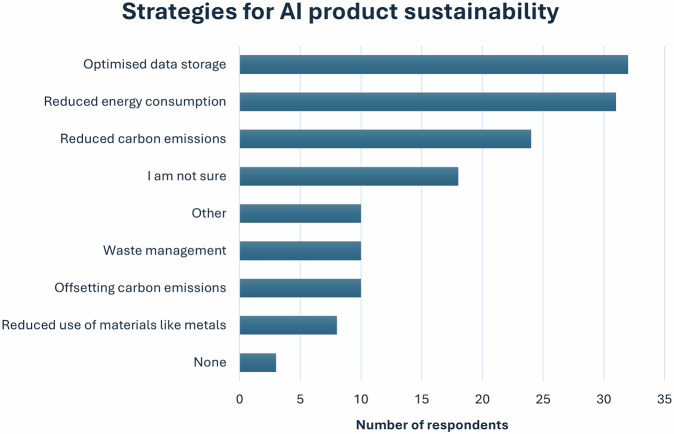
Steps taken to ensure AI product sustainability

The top 3 qualities towards responsible AI valued by the companies where the respondents (*n* = 69) worked were (a) data privacy/confidentiality (*n* = 46/69; 66.6%), (b) clinically led scope of practice (*n* = 29/69; 42%), and (c) fairness/diversity of data (*n* = 25/69; 36.2%). The above top-rated values were followed by robust governance frameworks (*n* = 20/69; 28.9%), transparency (*n* = 18/69; 26.1%), human-in-the-loop approaches (*n* = 16/69; 23.1%), explainability (*n* = 15/69; 21.7%), teamwork (*n* = 15/69; 21.7%), sustainability (*n* = 7/69; 10.1%), and team training (*n* = 6/69; 8.7%). AI industry employees (*n* = 71) also described steps that their companies took to ensure AI product sustainability (Fig. 3).

**Fig. 4:**
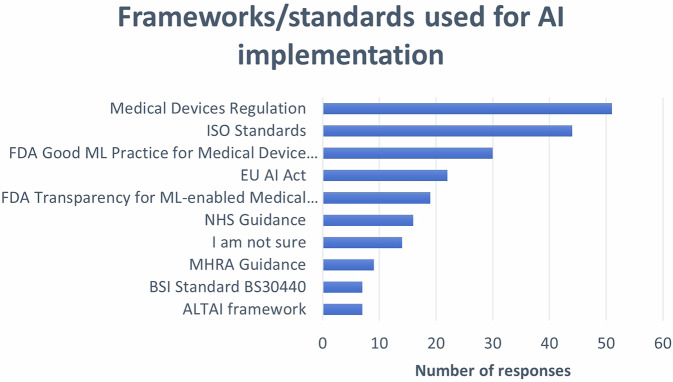
AI frameworks are used for AI development and deployment. * Multiple responses received for some options in this question

Study participants responded as to whether their company used AI-enabled tools in their business practice, e.g. for training, marketing, recruitment, etc. Almost half of them (*n* = 33/74; 44.6%) confirmed this practice, followed by those who were unsure about it (*n* = 21/74; 28.4%), and those who answered negatively (*n* = 19/74; 25.7%). In addition, another respondent (*n* = 1/74; 1.3%) clarified that AI use was evolving in order to be integrated into daily work. Among those who used AI-enabled tools in their daily practice, workflow automation and efficiency were the most prevalent areas of application (*n* = 19/26; 73.1%), followed by AI tools used for training purposes (*n* = 3/26; 11.5%), and those used for imaging applications (*n* = 2/26; 7.7%) or tendering purposes (*n* = 2/26; 7.7%). With regards to AI governance, the respondents (*n* = 69) reported frameworks or standards that their company used for the development and deployment of AI tools (Fig. 4). Specific quotations from the respondents’ open-ended questions in relation to these topics can also be found in Supplementary Material 2.

### Challenges and enablers of AI deployment

**Fig. 5:**
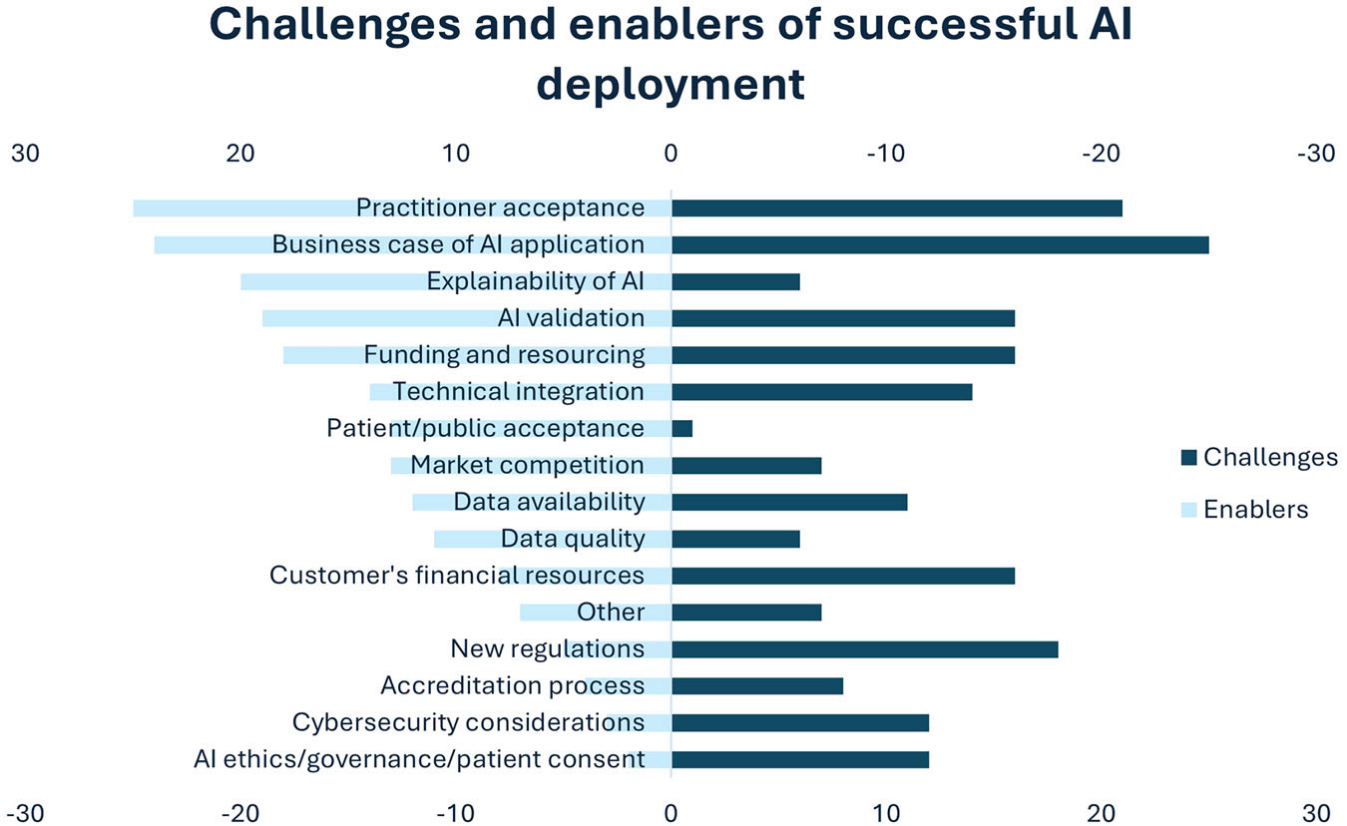
Enablers and challenges to successful AI deployment, as highlighted by study respondents, in descending frequency order (for enablers)

When it comes to the successful deployment of AI tools in clinical practice, different aspects were reported as enablers and challenges by the respondents (*n* = 65), as can be seen below (Fig. 5). The respondents recognised digital infrastructure issues (*n* = 41/64; 64.1%), lack of engagement from clinical settings (*n* = 35/64; 54.7%), and lack of financial resources (*n* = 27/64; 42.2%) as key issues preventing them from meeting their customer expectations, followed by lack of clear business cases (*n* = 22/64; 34.4%), complexity of clinical cases (*n* = 18/64; 28.1%), constantly changing regulations (*n* = 18/64; 28.1%), insufficient algorithm performance (*n* = 10/64; 15.6%), and lack of expertise within the company (*n* = 8/64; 12.5%).

Looking at the AI tool’s lifecycle, they reported that external testing (*n* = 37/62; 59.6%), post-market surveillance (*n* = 25/62; 40.3%), and AI deployment (*n* = 22/62; 35.4%) were the most difficult areas to address, followed by compliance with regulations (*n* = 20/62; 32.2%), co-production with end-users (*n* = 17/62; 27.4%), sustainability (*n* = 11/62; 17.7%), AI innovation (*n* = 9/62; 14.5%), and internal testing (*n* = 6/62; 9.6%).

**Fig. 6:**
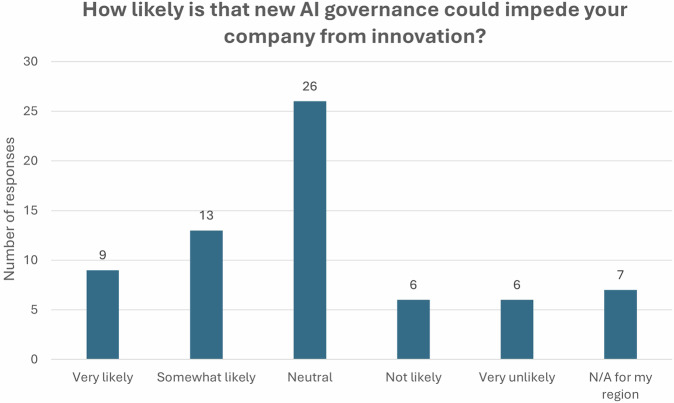
Self-referred potential impact of new AI governance on innovation

Figure 6 summarises the respondents’ views on whether new AI governance rules and regulations would impede their company from innovating. Many of them expressed neutral opinions about the impact of new AI governance on innovation, clarifying that the real impact of new regulation was imminent and placed potential consequences in the near future.

Respondents who believed that AI governance would stifle their company’s innovation, highlighted the additional workload that management of this regulation would add to their team, the associated costs, and the additional challenges that smaller companies may face, often disproportionate to larger ones. In addition, the respondents confirmed that since governance can be geographically variable, the innovation focus may be shifted to countries with less regulatory burden.

The respondents were asked to freely write their thoughts on the following statement: ‘Interoperability could increase operational efficiency, reduce costs, and enhance patient outcomes, but it can also stifle innovation’. The content analysis revealed that most of them (*n* = 31/50; 62%) disagreed that interoperability would stifle innovation in any way. However, some expressed neutrality (*n* = 10/50; 20%) or supported the opposite argument (*n* = 9/50; 18%).

Regarding opportunities or challenges arising from the use of generative AI in medical imaging, many of them expressed concerns about the impact of hallucinations and bias when it came to patient outcomes and diagnoses/prognoses (Table 2). They also noted that generative AI applications were currently limited in medical imaging, that they required rigorous audit and certification, and that serious validation challenges existed from a regulatory perspective. However, they stressed their optimism that generative AI has the potential to improve clinical workflows, reduce turnaround times of new products, and help mitigate workforce shortages.

**Table 2:** Challenges and opportunities arising from generative AI

Codes	Examples of key quotations	Frequencies of reference
Concerns about hallucinations and bias	“Ensuring the accuracy and reliability of generated images is critical, as inaccuracies could lead to misdiagnosis or inappropriate treatment plans”	*n* = 15 (39.4%)
Limited application at the present time	*“*Generative AI is still not sufficiently developed to be applicable as a medical device in any context”	*n* = 9 (23.6%)
Tools to improve clinical workflow	“Save time in repetitive tasks, help clinicians to make better diagnostics”	*n* = 8 (21%)
Potential for faster turnaround of new products	“Significant opportunities to generate training data in the future”	*n* = 3 (7.9%)
Needs rigorous audit and certification	“Not regulatory approved”	*n* = 2 (5.2%)
Hard to validate from a regulatory perspective	“Generative AI has a lot of promise, but it will be hard to validate and clear from a regulatory perspective”	*n* = 2 (5.2%)
Mitigate workforce shortages	“Generative AI presents opportunities to address the shortage of healthcare professionals and manage growing demand”	*n = 1 (2.6%)*
Potential for over-reliance and deskilling	“Can lead to over-reliance”	*n* = 1 (2.6%)

The respondents were asked to offer their perspectives on what would be needed to make AI deployment successful and sustainable. The results can be found in Tables 3 and 4, respectively.

**Table 3:** Requirements for successful AI deployment

Codes	Examples of key quotations	Frequencies of reference
Consistent funding	“Clear funding route for AI/medical imaging technology”	*n* = 11 (22.9%)
IT support within clinical departments	“Widening the IT bottleneck, both with (quality) people and physical infrastructure”	*n* = 9 (18.7%)
Clinician acceptance, user buy-in, and stakeholder engagement	“Senior stakeholder engagement”	*n* = 8 (16.6%)
Consensus standards or a global regulatory framework	“Clear and predictable regulatory framework on as large a geography as possible. Best—globally”	*n* = 7 (14.5%)
User training	“Educate medical professionals on the benefits and limitations of AI tools, providing training to ensure they are used effectively and ethically”	*n* = 7 (14.5%)
Clear business case and big-picture vision, and strategy	“A strong business case with real-world evidence and subsequent funding from hospitals to purchase AI”	*n* = 6 (12.5%)
Clinical AI champion for departmental link	“Key AI roles at hospital to support deployment”	*n* = 5 (10.4%)
Access to datasets	“Ensure access to high-quality, diverse, and annotated datasets to train and validate the AI models, adhering to privacy and ethical guidelines”	*n* = 4 (8.3%)
Empirical evidence of real-world testing	“Sufficient case examples to demonstrate robustness and recommended application of the product”	*n* = 4 (8.3%)
Transparency in testing	“National validation of algorithms performed transparently”	*n = 4 (8.3%)*
Global collaboration	“Cross domain/country/industry/generation/culture collaboration”	*n* = 3 (6.2%)
Supportive hospital infrastructure	“Better clinical settings, so it’s faster to include new technologies into their workflow”	*n* = 3 (6.2%)
Clinically useful and relevant tools	“AI, which is really solving problems”	*n* = 2 (4.1%)
Normalised use of AI in clinical settings	“Sufficient market of use”	*n *= 2 (4.1%)
Time for integration	“Time to implement”	*n* = 2 (4.1%)
Commercialisation and adoption pathway	“Clear commercialisation and adoption pathways”	*n* = 1 (2.1%)
Continuous monitoring	“Establish mechanisms for continuous monitoring of AI tool performance”	*n* = 1 (2.1%)
Efficient workflow integration	“Clinical workflow integration”	*n *= 1 (2.1%)
Faster product approval time	“Reducing the time it takes to get approval from organisations like the FDA, so products can get faster to the market”	*n* = 1 (2.1%)
Guidance for paid external consultancy	“Guidelines for radiologists taking paid external consultancies”	*n* = 1 (2.1%)
Recognition of PACS staff as project managers	“More recognition of the professional status of PACS and Imaging Informatics teams and expansion of them to include full-time technically aware Project Managers”	*n* = 1 (2.1%)

*PACS* Picture archiving and communication system

**Table 4:** Requirements for sustainable AI deployment

Codes	Examples of key quotations	Frequencies of reference
Optimise storage and GPU using cloud-based tools	“Use platform and cloud-enabled solutions to share resources and reduce hardware required”	*n* = 11 (30.5%)
Minimise carbon footprint	“Net zero data centres”	*n *= 5 (13.8%)
Clear strategy needed	“This needs to be part of a clear broader strategy. Local organisational level IT is far from sustainable, so expectations are still poor”	*n *= 4 (11.1%)
Funding support	“Regular long-term funding streams made available by the NHS”	*n* = 4 (11.1%)
Regulatory pathways	“Maintain strict adherence to ethical standards and regulatory requirements, updating protocols as guidelines evolve to ensure patient safety and data privacy”	*n* = 4 (11.1%)
Buy-in and collaboration for sustainable approaches	“Foster collaborations with technology providers, healthcare institutions, and academic research groups to drive innovation and share expertise”	*n* = 3 (8.3%)
Cost-efficacy ensured	“Ensure that the deployment of AI tools is economically viable by demonstrating cost-effectiveness through reduced diagnostic errors, optimised treatment plans, and improved patient throughput”	*n* = 3 (8.3%)
Global access to diverse data and data sharing	“A better pipeline of globally diverse data”	*n* = 2 (5.5%)
Health and accuracy outcomes prioritised over sustainability	“Focus should be on local quality improvement and other metrics that are equally and often more important for AI adoption”	*n* = 2 (5.5%)
Integration with existing hospital systems	“A good integration with the informatic system of the hospital”	*n* = 2 (5.5%)
Multi-tenancy applications	“Multi-tenancy applications”	*n* = 2 (5.5%)
Research and education	“NHS Publications on the ROI of AI. NHS Publications on the clinical utility of AI at scale. University-level education for Radiographers and Medics on the ethical use of AI”	*n* = 1 (2.7%)

*GPU* Graphics processing unit

## Discussion

This study highlights several critical factors influencing the successful development and deployment of AI in medical imaging and oncology, demonstrates the complexities of AI adoption within multidisciplinary teams and the importance of strategic collaboration between industry, regulators, and clinical stakeholders.

The findings of this study are supported by similar outcomes reported in other studies. First, this study shows the importance of co-production, with many respondents emphasising the need to involve both practitioners and end-users in AI product development. Co-production is a valuable practice for user-friendly and person-centred design in healthcare [20]. But, as our data shows can be mostly afforded by larger companies, as it is a resource-intensive activity. The same accounts for the provision of employee training. Furthermore, our data show that smaller companies may be more impacted by new governance requirements, which might impose initiatives requiring larger budgets.

Multidisciplinary teams should be empowered to guide the creation and design of AI technologies, led by real clinical problems [21]. Multidisciplinary and multiagency teams are vital to guide successful AI adoption and establish co-ownership of the tool [22, 23]. However, recent findings suggest that industry representatives are amongst the most under-represented groups of stakeholders in studies assessing the impact of AI, underlining the importance and timeliness of this study [24]. Including the AI vendors’ perspectives in all stages of AI implementation and fostering collaborative relationships may garner trust and support better working relationships, thus enhancing practitioner acceptance, a crucial enabler for AI deployment. These findings align with wider research showing that practitioner acceptance is vital for AI adoption, and a lack of acceptance represents a significant barrier to implementation [25, 26]. When it comes to responsible AI implementation, this study, like others before [27, 28], confirms the key role of reliability and transparency in fostering trust among end-users.

The fundamental role of appropriate reimbursement and the challenge of establishing a clear business case for AI tools are also highlighted. Many vendors struggle with securing financial resources and demonstrating the long-term economic value of their products, which poses a significant barrier to widespread and longer-term adoption, which is exactly where the real benefits can be seen. A previous survey among AI vendors stressed exactly this need for funding availability in different regions and systems [29]. Similarly, the lack of dedicated funding from clinical settings is an important barrier to AI adoption [10, 27]. When creating a business case, different departments within the clinical settings often need to collaborate and align their objectives, since the true benefits of adopting an AI tool won’t often be seen by the adopting department, but can rather be associated with clinical or other outcomes of a different department. A holistic evaluation of the benefits of AI is therefore required, recognising the interconnectivity of services in healthcare settings, with involvement of the senior and executive leadership within each organisation. This was confirmed by other studies, which showed that all AI tools must have well-defined business cases and follow rigorous evaluation processes [30]. A value-based approach ensures that AI tools have a clinically relevant impact on care. Health technology assessments could be employed to evaluate whether AI tools are beneficial to healthcare organisations [31].

Regulatory requirements represent a significant challenge around AI implementation, particularly because of the newly published EU AI Act and pending UK AI Bill [12, 32, 33]. Regulatory compliance emerged as a major concern for vendors from our data, particularly for those working at smaller enterprises, which often face disproportionate resourcing challenges in meeting evolving governance requirements. Developers need to be aware of the complexities associated with AI adoption, and regulators should be proactive in establishing standards [34] and collaborating with clinical practitioners and industry for meaningful and focused governance, that addresses grassroots challenges [35].

External testing of algorithms and post-market surveillance of AI tools were considered as the most challenging areas to address from the respondents’ perspective. This might relate to the complexity and the cost of these processes and the need for information exchange and collaboration with clinical clients, often impeded by data sharing and data privacy restrictions [35]. Furthermore, post-market surveillance can be complex and convoluted, as it may need to take into account changes in relation to local staffing, equipment and institutional processes [22]. It is, though vital, as a guard rail ensuring the stability of AI models’ performance despite changes in the clinical setting [36]. In addition, developers should be aware of any updated post-market surveillance regulatory requirements [35], and also possible live feedback from clients/users regarding needs for required updates and changes to their original AI tool for optimising patient care and contextualising to end-users in each clinical setting.

The findings of this study also revealed a significant overlap between key factors (e.g. practitioner acceptance, AI validation, business case of AI application) that largely appear both as challenges and enablers of successful AI deployment, as reported by AI vendors. This highlights the heterogeneity of the respondents’ previous experiences with AI implementation, which can also be attributed to differences in their professional backgrounds, years of experience, use cases where AI was deployed, clinical setting infrastructures, and local culture. Similar heterogeneities have been reported in previous studies [13, 37, 38], where different professions within medical imaging reported different priorities, needs, and challenges for a successful AI implementation. A recent study has also highlighted the limitations of AI deployment based on model setup, local infrastructure, and human factors [39].

### Limitations

Although responses were received from more than 100 respondents of different companies worldwide, this sample may not be representative of the entire AI vendor landscape. Confidentiality clauses, local governance and company policies might have restricted the sample size. This shows that there is more work to be done to build trust between end-users and industry and forge stronger collaborations. Moreover, the anonymous nature of the survey design, as required to encourage participation, might have prevented accurate identification of the number of independent vendors who participated or the number of respondents working per vendor or company. This limitation was acknowledged at the outset; however, it was decided that anonymity was essential to encourage participation, authenticity of responses, and honesty without risking customer relations or business dynamics. The geographical distribution of the respondents was widespread, albeit potentially more Europe and North America-focused, which could limit the generalisability of the findings to other regions, such as Asia or Africa. Future research should aim to recruit a larger sample over an extended period, to help identify whether the challenges and enablers of AI deployment differ, particularly given the changing governance landscape. The approach of convenience sampling through the networks of the collaborating researchers of this study might have also introduced self-selection bias, and this should be seen as an additional limitation. Finally, despite our best intentions to run inferential statistics, the small number of responses in the different sub-groups we wanted to examine, did not allow us to perform a reliable statistical analysis.

In conclusion, people, infrastructure and funding models remain at the core of AI implementation, both as challenges and enabling factors. AI vendors must be prepared to face the new challenges coming with new governance and legislation, which will inevitably pose more pressure on small and medium-sized enterprises. Employing responsible practices, such as codesigning with end-users, ensuring sustainability and transparency are values universally appreciated by practitioners and industry alike, and are key determinants of strong clinical-industry collaborations and successful AI implementation programmes.

## Supplementary information

Supplementary information

## Abbreviations

AI: Artificial intelligence
EU: European Union
FDA: Food & Drug Administration
IT: Information technology
NHS: National Health Service
SPSS: Statistical package for social sciences
UK: United Kingdom
