# Supplementary material for: Vendors’ perspectives on AI implementation in medical imaging and oncology: a cross-sectional survey
Source: Eur Radiol. 2025 Sep 23;36(3):1686–97. doi: 10.1007/s00330-025-12013-1 (PMC12963099; doi:10.1007/s00330-025-12013-1)
Supplement: Supplementary file 1 — Supplementary information [file 330_2025_12013_MOESM1_ESM.pdf]

Vendors’ perspectives on AI implementation in medical imaging and oncology: a cross-sectional survey

ELECTRONIC SUPPLEMENTARY MATERIAL

Note: Any reuse or reproduction of the survey by others will require discussion with and permission by the study’s senior (last) author.

Demographic data

1.Does your organisation develop or distribute FDA/CE/UKCA accredited AI-enabled tools for use in medical imaging and/or radiation therapy?

- ◯ Yes
- ◯ No

2.Please specify your age range. Check the option that applies from the dropdown menu below.

- ◯ 18-29 years old
- ◯ 30-39 years old
- ◯ 40-49 years old
- ◯ 50-59 years old
- ◯ 60-69 years old
- ◯ 70+ years old

3.Please use the box provided to define the gender you identify with:

- ◯ Male
- ◯ Female
- ◯ Non-binary
- ◯ I do not want to disclose
- ◯ Please use the box provided to define the gender you identify with: ____________________

4.What is your professional background?

- ◯ Business/Management
- ◯ Engineering
- ◯ Informatics
- ◯ Clinical practitioner (e.g., radiologist, radiographer etc.). Please explain: ____________________
- ◯ Other. Please explain: ____________________

5.What is your current role? Please choose up to 2 options all that apply from the below list.

- ☐ Education Lead
- ☐ Clinical Applications Specialist
- ☐ R&D Team
- ☐ Marketing Team
- ☐ Sales Representative
- ☐ Senior Executive
- ☐ Innovation Lead
- ☐ Scientific Affairs
- ☐ Regulatory or Quality Affairs
- ☐ Other. Please explain: ____

6.How many years' experience do you have in the above role?

- ◯ 0-10 years
- ◯ 11-20 years
- ◯ 21-30 years
- ◯ >30 years

7.What is your career level currently?

- ◯ Early career
- ◯ Mid-career
- ◯ Advanced
- ◯ Other. Please explain: ____

8.Did you have any AI-related training/education?

- ◯ Yes. Please explain: ____
- ◯ No
- ◯ Other. Please explain: ____

9.If yes, what kind of training/education?

- ☐ Education provided by a University
- ☐ Hands-on training at conferences/seminars/workshops
- ☐ Training provided by my organisation
- ☐ Training provided by professional bodies
- ☐ Self-guided training/reading
- ☐ Other. Please explain: ______________________________________

Company data

10.Please do not name your company, but we would like to know what kind of company you work for.

- ◯ Micro enterprise (1-9 employees)
- ◯ Small enterprise (10-49 employees)
- ◯ Medium-sized (50-249 employees)
- ◯ Large enterprise (>250 employees)
- ◯ Other. Please elaborate: ____

11.Which generic geographical location is the company you work at mainly operating at currently? Please choose all options that apply.

- ☐ North America
- ☐ South America
- ☐ Europe
- ☐ Africa
- ☐ Middle East
- ☐ Asia
- ☐ Oceania

12.Does your company provide AI training for employees (e.g., about algorithms, or about the AI market, or about regulatory affairs around AI, or about your own AI products)?

- ◯ Yes. Please elaborate: ____
- ◯ No
- ◯ Other. Please explain: ____

13.What kind of AI tools does your company develop/market? Please choose all options that apply.

- ☐ Workflow
- ☐ Triage
- ☐ Detection
- ☐ AI-assisted diagnosis
- ☐ Image quality enhancement
- ☐ Radiation dose reduction
- ☐ Image analysis enhancement
- ☐ Safety optimisation
- ☐ Patient care optimisation
- ☐ Other. Please explain: ____

14.Does your organisation use AI-enabled tools in daily routine, e.g. for training, workflows, marketing, recruitment?

- ○ Yes. Please explain in what context: ____
- ○ No
- ○ I am not sure
- ○ Other. Please explain: ____

15.Do you employ co-production/co-creation* of AI tools with end users in your company?
**Producing or creating AI-enabled solutions with the end users working as equal partners with*

the rest of the team, to ensure functionality, user-friendliness, applicability of AI solutions. End users could be healthcare professionals, patients, their carers, or the public.

- ○ Yes. Please explain in what way you involve users: ___________________________
- ○ No. Please explain why you do not involve users: ______________________________
- ○ I am not sure
- ○ Other. Please explain: ____

AI implementation

16.What active steps does your company take to ensure AI product sustainability*? Please choose up to 3 from the list below. **e.g., reduced carbon emissions and optimised energy consumption, data storage, and waste generation.*

- ☐ Reduced energy consumption
- ☐ Reduced use of materials like metals or helium
- ☐ Optimised data storage
- ☐ Waste management
- ☐ Reduced carbon emissions
- ☐ Offsetting carbon emissions
- ☐ I am not sure
- ☐ Other. Please explain: ____
- ☐ None

17.What qualities does your company mostly value to ensure responsible AI? Please choose **your top 3** from below:

- ☐ Transparency
- ☐ Explainability
- ☐ Fairness/diversity of training data
- ☐ Data privacy/confidentiality
- ☐ Clinically led scope of practice
- ☐ Sustainability
- ☐ Human in the loop
- ☐ Teamwork/collaboration
- ☐ Team training/AI education
- ☐ Robust governance frameworks, e.g., for liability/accountability
- ☐ Other, please explain: ____

18.What frameworks or standards does your company use for the development and deployment of AI tools? Please choose all options that apply.

- ☐ Medical Devices Regulation. Please explain: ______
- ☐ ISO standards. Please explain: ______
- ☐ NHS guidance. Please explain: ______
- ☐ MHRA guidance. Please explain: ______
- ☐ BSI Standard BS30440. Please explain: ______
- ☐ Other. Please explain: ______
- ☐ EU AI Act
- ☐ ALTAI ramework (EU) https://digital-strategy.ec.europa.eu/en/library/assessment-list-trustworthy-artificial-intelligence-altai-self-assessment
- ☐ FDA Good Machine Learning Practice for Medical Device Development: Guding Principles https://www.fda.gov/medical-devices/software-medical-device-samd/good-machine-learning-practice-medical-device-development-guiding-principles
- ☐ FDA Transparency for Machine Learning-Enabled Medical Devices: Guiding Principles https://www.fda.gov/medical-devices/software-medical-device-samd/transparency-machine-learning-enabled-medical-devices-guiding-principles
- ☐ I am not sure

19.How likely is that new aspects of AI governance (e.g. UK AI Bill, EU AI Act) would impede you/your company from innovation?

- ◯ Very likely. Please explain: ____________________
- ◯ Somewhat likely. Please explain: ____________________
- ◯ Neutral. Please explain: ____________________
- ◯ Not likely. Please explain: ____________________
- ◯ Very unlikely. Please explain: ____________________
- ◯ Not applicable for my region

20.Please write your thoughts on the following statement: ''Interoperability* could increase operational efficiency, reduce costs, and enhance patient outcomes, but it can also stifle innovation''. **Interoperability refers to the standards, protocols, technologies, and mechanisms that allow data to flow between diverse systems with minimal human intervention. It allows diverse systems to talk to each other and share information in real time.*

____

21.Which of the following do you recognise as challenges when it comes to the deployment of AI tools in clinical practice? Please choose your **top 3** from the below options.

- ☐ (New) Regulations (EU AI Act, UK AI Bill etc)
- ☐ Accreditation process
- ☐ AI validation
- ☐ Market competition
- ☐ Funding and resourcing
- ☐ Cybersecurity considerations
- ☐ AI ethics/governance/patient consent
- ☐ Explainability of AI
- ☐ Data availability
- ☐ Data quality
- ☐ Practitioner acceptance
- ☐ Patient/public acceptance
- ☐ Technical integration
- ☐ Business case of AI application/return on investment
- ☐ Customer's financial resources
- ☐ Other. Please explain: ____

22.Which of the following do you recognise as enablers when it comes to the deployment of AI tools in clinical practice? Please choose **your top 3** from the below options:

- ☐ (New) Regulations (EU AI Act, UK AI Bill etc)
- ☐ Accreditation process
- ☐ AI validation
- ☐ Market competition
- ☐ Funding and resourcing
- ☐ Cybersecurity considerations
- ☐ AI ethics/governance/patient consent
- ☐ Explainability of AI
- ☐ Data availability
- ☐ Data quality
- ☐ Practitioner acceptance
- ☐ Patient/public acceptance
- ☐ Technical integration
- ☐ Business case of AI application/return on investment
- ☐ Customer's financial resources
- ☐ Other. Please explain: ____

23.Which of the following may impede your AI products from meeting customer expectations? Please choose **your top 3** from the below options:

- ☐ Lack of expertise within the company
- ☐ Complexitiy of clinical cases
- ☐ Lack of financial resources
- ☐ Insufficient algorithm performance
- ☐ Infrastructure issues at clinical settings
- ☐ Constantly changing regulations
- ☐ Lack of engagement from clinical settings
- ☐ Lack of clear business case for our solution
- ☐ Other. Please explain: ____

24.Which area of an AI product's lifecycle do you find most difficult to address? Please choose **your top 3** from the below options:

- ☐ AI innovation
- ☐ Co-production/co-creation with end users
- ☐ Compliance with regulations
- ☐ Internal validation
- ☐ External validation
- ☐ AI deployment
- ☐ Post-market surveillance

☐ Sustainability

☐ Other. Please explain: ____

25.Please explain what you would need to make the deployment of AI tools in medical imaging/radiotherapy **successful**: ____

26.Please explain what you would need to make the deployment of AI tools in medical imaging/radiotherapy **sustainable:** ____

27.What opportunities or challenges do you see from Generative AI in the context of medical imaging and/or radiation therapy? Please explain: _______________________________

28.Please add any further comments/ideas on what industry needs to make AI happen in clinical practice: ____

Representative quotes from participants

Co-production with end-users:

‘Clinical users have been involved since the inception of the company, and we remain driven by clinical priorities’

Co-production as part of user testing:

‘In any new release of the product (that involves AI features), external collaborators and customers are encouraged to give us feedback on the user-friendliness and functionality in clinical setting of any addition to the product’

Impact of new AI governance on innovation:

‘Progression of AI in the UK is already stifled by fragmented strategies and governance in place. It is too early to know whether these Bills will help or hinder’

‘I think there is rightly greater focus on risks of bias and importance of diversity, but these things can be challenging to address for a small company with limited access to clinical data and limited budget for prospective data acquisition’

‘It is important that we operate within all regulatory and governance frameworks’

Impact of interoperability on innovation:

‘Not sure why "Interoperability" can stifle innovation. Once a system is interoperable with others and allows a seamless workflow, it can foster innovation, creating AI solutions that can be integrated in the standard of care and clinical routine in an easy and straight forward way’

‘By creating a rigid framework within which developers must operate, these standards can also limit how developers build their software, potentially discouraging exploration of novel approaches that don't fit within the established parameters. This could slow down the introduction of groundbreaking technologies that might initially seem incompatible with existing systems. Overly restrictive standards might prioritize compatibility over innovation, leading to a technological landscape that evolves more slowly’

Opportunities and challenges arising from Generative AI:

‘Ensuring the accuracy and reliability of generated images is critical, as inaccuracies could lead to misdiagnosis or inappropriate treatment plans’

‘Generative AI has a lot of promise but will be hard to validate and clear from a regulatory perspective’
